# Production of Functionally Active and Immunogenic Non-Glycosylated Protective Antigen from *Bacillus anthracis* in *Nicotiana benthamiana* by Co-Expression with Peptide-N-Glycosidase F (PNGase F) of *Flavobacterium meningosepticum*

**DOI:** 10.1371/journal.pone.0153956

**Published:** 2016-04-21

**Authors:** Tarlan Mamedov, Jessica A. Chichester, R. Mark Jones, Ananya Ghosh, Megan V. Coffin, Kristina Herschbach, Alexey I. Prokhnevsky, Stephen J. Streatfield, Vidadi Yusibov

**Affiliations:** Fraunhofer USA Center for Molecular Biotechnology, Newark, Delaware, United States of America; Van Andel Research Institute, UNITED STATES

## Abstract

*Bacillus anthracis* has long been considered a potential biological warfare agent, and therefore, there is a need for a safe, low-cost and highly efficient anthrax vaccine with demonstrated long-term stability for mass vaccination in case of an emergency. Many efforts have been made towards developing an anthrax vaccine based on recombinant protective antigen (rPA) of *B*. *anthracis*, a key component of the anthrax toxin, produced using different expression systems. Plants represent a promising recombinant protein production platform due to their relatively low cost, rapid scalability and favorable safety profile. Previous studies have shown that full-length rPA produced in *Nicotiana benthamiana* (pp-PA83) is immunogenic and can provide full protection against lethal spore challenge; however, further improvement in the potency and stability of the vaccine candidate is necessary. PA of *B*. *anthracis* is not a glycoprotein in its native host; however, this protein contains potential N-linked glycosylation sites, which can be aberrantly glycosylated during expression in eukaryotic systems including plants. This glycosylation could affect the availability of certain key epitopes either due to masking or misfolding of the protein. Therefore, a non-glycosylated form of pp-PA83 was engineered and produced in *N*. *benthamiana* using an *in vivo* deglycosylation approach based on co-expression of peptide-N-glycosidase F (PNGase F) from *Flavobacterium meningosepticum*. For comparison, versions of pp-PA83 containing point mutations in six potential N-glycosylation sites were also engineered and expressed in *N*. *benthamiana*. The *in vivo* deglycosylated pp-PA83 (pp-dPA83) was shown to have *in vitro* activity, in contrast to glycosylated pp-PA83, and to induce significantly higher levels of toxin-neutralizing antibody responses in mice compared with glycosylated pp-PA83, *in vitro* deglycosylated pp-PA83 or the mutated versions of pp-PA83. These results suggest that pp-dPA83 may offer advantages in terms of dose sparing and enhanced immunogenicity as a promising candidate for a safe, effective and low-cost subunit vaccine against anthrax.

## Introduction

Anthrax is an acute disease caused by the bacterium *Bacillus anthracis*, affecting both humans and animals. Of the three forms of the disease caused by *B*. *anthracis*–cutaneous, gastrointestinal and inhalational–the disease caused by inhalation of aerosolized spores is the most severe with the highest mortality rate of about 86–89% [1]. *B*. *anthracis* spores are relatively easy to produce and release and thus, can be used by bioterrorists, as was evidenced by the 2001 incidences of spore-containing letter attacks in the U.S. *B*. *anthracis* secretes three toxin proteins: edema factor (EF, a calmodulin-dependent adenylate cyclase), lethal factor (LF, a metalloprotease), and protective antigen (PA) that act in binary combinations to form two AB-type toxins, the edema toxin (ET = PA+EF) and the lethal toxin (LeTx = PA+LF). After binding to the cell surface, PA is proteolytically cleaved by furin, which results in the release of a 20-kDa protein fragment and heptamerization of 63-kDa fragments to form a pre-pore [2]. Heptamerized PA binds LF or EF and facilitates the exotoxin entry into the cytoplasm, leading to cell death.

Currently, Anthrax Vaccine Adsorbed (BioThrax^®^), licensed in 1972, is the only U.S. Food and Drug Administration (FDA)-licensed human anthrax vaccine in the U.S. The vaccine contains the 83-kDa PA protein prepared from cell-free filtrates of microaerophilic cultures of an avirulent, non-encapsulated strain of *B*. *anthracis*, adsorbed to aluminum hydroxide gel as an adjuvant. The main drawbacks of BioThrax are that the vaccine may contain other anthrax proteins, including LF and EF, which can potentially cause severe allergic reactions, including anaphylaxis and even death. In addition, the vaccine induces only limited protection and requires a lengthy course of administration to achieve protective immunity [3,4]. These drawbacks have led to increased efforts in recent years to develop new, second-generation anthrax vaccines, including recombinant live and recombinant subunit vaccines [5–11]. The most advanced vaccine candidates are based on recombinant PA (rPA) expressed in and purified from *Escherichia coli* [5], or PA prepared from an asporogenic, non-toxigenic, non-encapsulated strain of *B*. *anthracis* [6,7]. rPA-based vaccines have been shown to induce high-titers of anti-PA toxin-neutralizing antibody (TNA) responses in animals and protect rabbits and non-human primates against lethal *B*. *anthracis* challenge [12,13]; however, in some studies protection waned dramatically over 6 to 12 months [13], indicating a need for vaccine formulations that can induce stronger, more robust long-lasting immunity.

Advances in heterologous expression have triggered an interest in using plants as an alternative platform for the production of recombinant proteins including subunit rPA-based vaccine candidates. Plants have perceived safety advantages as they do not harbor mammalian pathogens and cost and scalability advantages as stainless steel fermenters are not required. In addition, plant cells perform eukaryotic post-translational modifications of target proteins, including N-linked glycosylation, which are substantially similar to those found in mammalian cells [14]. Although rPA contains six potential N-linked glycosylation sites, it is not glycosylated in its native host. When expressed in *N*. *benthamiana* plants, however, rPA is glycosylated. As a result, this glycosylated rPA molecule elicited TNA titers in mice, but could not form LeTx *in vitro* [15]. We hypothesized that this may be a result of N-glycosylation acquired in the plant host and that the presence of these sugars has a negative impact on the stability and potency of rPA, two desired attributes of a safe and effective vaccine.

Recently, we have developed a strategy of enzymatic deglycosylation of proteins *in planta* by co-expressing bacterial peptide-N-glycosidase F (PNGase F) from *Flavobacterium meningosepticum* with target protein [16]. Our studies have demonstrated that enzymatic deglycosylation of target proteins *in vivo* by PNGase F has the potential to become a robust strategy for production of non-glycosylated proteins in plants. Here, the PNGase F-based *in vivo* deglycosylation approach has been applied towards producing a non-glycosylated form of pp-PA83 (pp-dPA83). Unlike glycosylated pp-PA83, pp-dPA83 is biologically active at levels comparable to the native prokaryotic form, indicating the great potential to be a target for a safe, effective, low-cost, second-generation vaccine development against anthrax. We also explored a site-directed mutagenesis-based approach and compared properties of the resulting pp-PA83 deglycosylated mutants to those of pp-dPA83.

## Materials and Methods

### Construction and Co-expression of pp-PA83 and PNGase F

Glycosylated pp-PA83 was produced using pGRD4, a Tobacco mosaic virus-based expression vector [17,18], into which the sequence encoding PA83 was sub-cloned to generate pGRD4-PA83. To produce pp-dPA83, sequences of pp-PA83 and PNGase F were cloned into the Beet yellows virus (BYV)-based miniBYV vector capable of co-expressing two functionally active recombinant proteins within the same host cell [19], under the control of the BYV coat protein (CP) promoter and the Grapevine leaf roll associated virus CP promoter. All genes were optimized for expression in *N*. *benthamiana* plants (for codon optimization, mRNA stability, etc.) and synthesized by GeneArt^®^ AG (Regensburg, Germany) as described previously [16]. The optimized pp-PA83 and PNGase F were engineered to contain the N-terminal tobacco pathogenesis-related protein 1a signal peptide (MGFVLFSQLPSFLLVSTLLLFLVISHSCRA, cleaved during protein secretion) and the C-terminal poly-histidine (6×His) affinity purification tag (FLAG was added to PNGase F) and the endoplasmic reticulum retention signal KDEL. The resulting construct was introduced into *Agrobacterium tumefaciens* strain GV3101, and plant infiltration was performed as described elsewhere [18,20].

### Engineering of pp-PA83 Mutants

Two mutant versions of pp-PA83 were designed based on results of liquid chromatography-tandem mass spectrometry (LC/MS/MS) of full-length pp-PA83 [15]. pp-PA83 has nine potential N-linked glycosylation sites [15] where asparagine is glycosylated at the consensus asparagine-X-serine/threonine (NXS/T) motif [21]. Glycosylation profiling by LC/MS/MS determined that six out of nine sites have some level of glycosylation (Asn-275, 357, 417, 505, 599 and 693) [15]. Thus, two mutant versions of pp-PA83, pp-PA83M-D (N275D, N357D, N417D, N505D, N599D and N693D) and pp-PA83M-Q (N275Q, N357Q, N417Q, N505Q, N599Q and N693Q), were synthesized by GeneArt^®^ with *PacI* and *XhoI* sites. The sequences of the pp-PA83 mutants were optimized for expression in *N*. *benthamiana* and inserted into the pGRD4 expression vector. The plasmids were then introduced into *A*. *tumefaciens* strain GV3101, and plant infiltration was performed as described elsewhere [18,20].

### Purification of Glycosylated and Non-glycosylated pp-PA83 Proteins from *N*. *benthamiana*

For glycosylated pp-PA83, at seven days post infiltration, leaf tissues from infiltrated plants were harvested, homogenized and extracted in a phosphate-based buffer. The extract was clarified by centrifugation and microfiltration, and pp-PA83 was purified as described previously [15].

Deglycosylated PA83, pp-dPA83, was purified in a similar manner as pp-PA83. Processing of pp-dPA83 was performed manually with a peristaltic pump and fractions were evaluated for total protein by Bradford analysis. Processing was performed at a scale of ~50 g of infiltrated biomass and utilized spin concentrators with dialysis. Fifty grams of infiltrated frozen plant leaves expressing pp-dPA83 were ground in a phosphate extraction buffer, clarified by centrifugation using JA 25.5 rotor for 25 minutes at 4°C at 20 K rpm, and the supernatant was passed through Miracloth prior to passage through a 0.45 μm Nalgene™ filter (Thermo Scientific, USA). The filtered supernatant was passed through the equilibrated (50 mM sodium phosphate, 0.5 M NaCl, 20 mM imidazole, pH 7.5) HisTrap FF 5 mL column (GE#17-5255-01) at a flow rate of 3 mL/min using a peristaltic pump. The column was washed with 8 column volumes (CV) of equilibration buffer and eluted with 8 CV of elution buffer (50 mM sodium phosphate, 0.5 M NaCl, 150 mM imidazole, pH 7.5). Total protein was evaluated in the elution fractions using the Bradford assay. Fractions containing protein (~3 CV) were combined and concentrated with a 10k MWCO Millipore concentrator to a final volume of 2 mL and buffer-exchanged against phosphate buffered saline (PBS). The concentrated protein was stored overnight in –20°C. The next day, the concentrated protein was thawed and centrifuged at 13.5 x g for 4 minutes at 4°C. Ammonium sulfate powder was added to the supernatant to a final concentration of 1 M, and the supernatant was passed through an equilibrated (PBS, 1 M ammonium sulfate) HiTrap phenyl HP 1 mL column (GE#17-1351-01) at a rate of 0.8 mL/min. The column was then washed with 8 CV of equilibration buffer and eluted with 8 CV of elution buffer (PBS, 0.6 M ammonium sulfate). After estimating total protein content using the Bradford assay, elution fractions (~5 CV) were combined, concentrated to 500 μL and buffer-exchanged against 20 mM sodium phosphate, pH 7.5. The buffer-exchanged fraction was then passed through a HiTrap CaptoQ FF 1 mL column (GE#11-0013-02) equilibrated with 20 mM sodium phosphate, pH 7.5 at a flow rate of 0.8 mL/min. The column was then washed with 8 CV of equilibration buffer and eluted with 8 CV of elution buffer (20 mM sodium phosphate, 120 mM NaCl, pH 7.5). After estimating total protein content using the Bradford assay, the elution fractions were combined (~4 CV), concentrated to 250 μL and buffer-exchanged against PBS. The purified protein was analyzed using sodium dodecyl sulfate polyacrylamide gel electrophoresis (SDS-PAGE) with bovine serum albumin (BSA) as a standard. The protein was aliquoted and stored in –80°C.

pp-PA83 mutant proteins (pp-PA83M-D and pp-PA83M-Q) were purified from *N*. *benthamiana* using a similar protocol to that for purification of pp-dPA83.

*In vitro* deglycosylated pp-PA83 was obtained by *in vitro* treatment of purified pp-PA83 with commercial PNGase F (Cat. No. P0704L, New England Biolabs, Ipswich, MA) according to the manufacturer’s instructions for the non-denaturing reaction conditions.

In order to minimize processing differences, additional preparations of pp-PA83 and the *in vivo* deglycosylated protein (pp-dPA83) for a second immunogenicity study were made using a more closely matched purification method. The modified purification procedure utilized 200 g of infiltrated biomass, which was processed through immobilized metal affinity chromatography (IMAC) in a similar manner as described above. After elution from IMAC, the samples were dialyzed into 20 mM Tris/5 mM phosphate buffer and polished over a diethylaminoethyl-Sepharose Fast Flow (DEAE FF) column (GE #17-0709-01). pp-PA83 was eluted from DEAE with 70 mM NaCl, while pp-dPA83 was eluted with 80 mM salt. Both proteins were concentrated and loaded directly onto a Superdex S-200 column (GE# 17-1043-02) and the main peak was collected, concentrated and filtered through a 0.22 μm cellulose acetate syringe filter. Final, filtered samples were aliquoted and stored frozen at ≤ -70°C.

### Stability Assessment

pp-PA83 versions were diluted to 0.5 mg/mL, aliquoted into low-retention, polypropylene Eppendorf tubes, and incubated at 37°C for 1 hour or at 4°C for 72 hours. Samples were taken immediately prior to and after incubation and mixed with SDS loading dye. Samples were stored frozen at -20°C until analysis by SDS-PAGE.

### SDS-PAGE and Western Blot Analysis

SDS-PAGE analysis of pp-PA83 or pp-dPA83 samples was performed on 10% acrylamide gels stained with Coomassie (Gel Code Blue, Pierce Rockford, IL). Western blot analysis was performed after electrophoresis and transfer of the proteins to Polyvinylidene Fluoride membranes. After transfer, Western blot membranes were blocked with I-Block (Applied Biosystems, Carlsbad, CA) and recombinant proteins detected with an anti-4xHis monoclonal antibody (mAb) (Qiagen, Valencia, CA). The membranes were then washed with 1 x PBS containing 0.1% Tween 20 (PBS-T) to remove an excess primary antibody and labeled with an anti-mouse horseradish peroxidase (HRP)-conjugated secondary antibody (1:10,000 dilution; Jackson ImmunoResearch, West Grove, PA). Signal generation was achieved with a chemiluminescent substrate (SuperSignal West Pico, Thermo Fisher Scientific, Grand Island, NY). Final images were captured with either a flatbed scanner or a Genome chemiluminescence detector (Syngene Corp., Frederick, MD).

### Glycan Detection Analysis

The presence of glycans in purified pp-PA83 samples was evaluated using Pro-Q Emerald 300 glycoprotein staining as described previously [16]. Briefly, 0.25 μg of pp-PA83 protein was run on 10% SDS-PAGE followed by detection of glycans in the gel using the Pro-Q Emerald 300 glycoprotein staining kit (Cat. No. P33378, Molecular Probes, Grand Island, NY) according to the manufacturer’s protocol. The SDS-PAGE gels were visualized using ultraviolet (UV) illumination.

### ELISA and BLItz Analysis

To assess the binding affinity between pp-PA83 variants and a plant-produced non-glycosylated anti-PA mAb (pp-mAb^PANG^ [22]), enzyme-linked immunosorbent assay (ELISA) plates were coated with different concentrations of the purified PA83 proteins (1000, 500, 100, 50, 10, 5, 1, 0.5 and 0.1 ng/mL) overnight. Plates then were washed with PBS-T, and blocking buffer was added for 2 hours at room temperature. The primary antibody (pp-mAb^PANG^) was added at the concentration of 2.0 μg/mL, and the plates were incubated at room temperature with shaking for 2 hours. Plates were washed with PBS-T, and a secondary anti-human IgG antibody conjugated to HRP (Jackson ImmunoResearch, West Grove, PA) was added and incubated for 1 hour. An ELISA substrate (*o*-Phenylenediamine dihydrochloride) was added, and plates were read at optical density (OD) of 490 nm with 650 nm as a reference.

The equilibrium dissociation constants, K_D_, were determined using a bio-layer interferometer, the BLItz^®^ system (Pall Forte Bio Corp., Menlo Park, CA), which provides real-time data on protein-protein interactions. pp-mAb^PANG^ was bound at the concentration of 100 μg/mL to a protein A sensor, and the PA83 variants were bound to pp-mAb^PANG^ at concentrations of 10, 20 and 40 μg/mL. K_D_ values were calculated from the results of dissociation and association.

### Anthrax LeTx Forming Assay (TFA)

A mouse macrophage cell line (J774A.1 cells, ATCC TIB-67, Manassas, VA) were plated in 96-well flat-bottomed tissue culture plates at 2.5x10^4^ cells/well in 50 μL and incubated for 16–19 hours at 37°C with 5% CO_2_. pp-PA83 variants were titrated on the plated cells in the presence or absence of LF (List Biological Laboratories, Campbell, CA). After a 4 hour incubation at 37°C with 5% CO_2_, cell viability was accessed by adding WST-1 (Roche Applied Sciences, Indianapolis, IN), a proliferation reagent, followed by a spectrophotometric measurement at 450 nm. A four-parameter logistic-log regression model was used to analyze the OD versus the PA83 concentration. The inflection point for each curve from this model is reported as showed the effective concentration 50% (EC_50_) in ng/mL of PA83 for the corresponding protein sample.

### pp-PA83 Immunogenicity Studies in Mice

Immunogenicity of the pp-PA83 antigen variants were evaluated in mice. In this study, seven-week-old BALB/c mice (Envigo, Indianapolis, IN), 5 per group, were immunized intramuscularly (IM) on study days 0, 21 and 42 with 10 or 3 μg of pp-PA83 protein adsorbed to 0.3% Alhydrogel (Accurate Chemicals, Westbury, NY). Serum samples were collected on days -1 (pre-bleed), 20 (post 1^st^ vaccination), 42 (post 2^nd^ vaccination) and 56 (post 3^rd^ vaccination) and assessed for anti-PA antibody responses by an IgG ELISA and an anthrax LeTx neutralization assay.

In a second mouse immunogenicity study, eight-week old BALB/c mice, 5 mice per group, were immunized IM on study days 0 and 21 with either pp-PA83 or pp-dPA83. Two doses of antigen were evaluated, a 10 μg dose, with and without 0.3% Alhydrogel, and a 1 μg dose plus Alhydrogel. A group of mice immunized with PBS served as a negative control. Serum was collected from the animals on study day 42 and assessed for anti-PA antibody responses by ELISA and an anthrax LeTx neutralization assay.

All animal studies reported here were conducted in compliance with the Animal Welfare Act regulations in the *Guide for Care and Use of Laboratory Animals*. All procedures were approved by the Institutional Animal Care and Use Committee at the University of Delaware (IACUC; Newark, DE). Animals were housed in the Life Sciences Research Facility at the University of Delaware (Assurance number A3773-01) and maintained under Specific Pathogen Free (SPF) conditions, with routine monitoring to confirm their disease-free status. Mice were housed in autoclaved ventilated micro-isolator housing units supplied with High-Efficiency Particulate Arrestance (HEPA)-filtered air. They received filtered tap water and Certified Purina Rodent Chow *ad libitum*. Animals were observed in the cage for indications of toxicity or ill health daily by monitoring activity level, behavior, appearance, signs of respiratory distress, weight loss and food intake. None of the animals in this study exhibited any indication of infection or distress. As only momentary or no pain was anticipated upon immunization with the vaccine candidate, no anesthetics, analgesics or tranquilizers were used. The University of Delaware’s IACUC has adopted a double kill policy. In compliance with this policy, the primary method of euthanasia for mice was tank-supplied CO_2_ followed by cervical dislocation as a secondary method of euthanasia.

### Assessment of Serum Anti-PA IgG and TNA Antibody Titers

Serum anti-PA IgG was detected by ELISA as described previously [23], and data was expressed as the geometric mean titer (GMT) ± standard error (SE) per group. Briefly, serum samples were titrated on plates coated with commercially available PA (List Biological Laboratories). End point titers were defined as reciprocal serum dilutions that give a mean OD value three times greater than the pre-immune control samples.

TNA titers in sera of immunized mice were measured using a LeTx neutralization assay as described previously [24] with modifications. J774A.1 cells were plated in 96-well flat-bottomed tissue culture plates at 2.5x10^4^ cells/well in 50 μL and incubated for 16–19 hours at 37°C with 5% CO_2_. Serum samples were diluted and titrated on the plated cells in the presence or absence of LF (List Biological Laboratories). After a 4 hour incubation at 37°C with 5% CO_2_, cell viability was assessed by adding WST-1 followed by a spectrophotometric measurement at 450 nm. A four-parameter logistic-log regression model was used to analyze the OD versus the reciprocal of the serum dilution. The inflection point for each curve from this model is reported as effective dose 50% (ED_50_) for the corresponding serum sample. The geometric mean of the ED_50_ values per group of animals were also calculated. Statistical significance was calculated using the one-way ANOVA and Kruskal-Wallis tests.

## Results

### Production of Non-glycosylated pp-PA83 in *N*. *benthamiana* by Enzymatic or Mutagenic Approaches

In this study, non-glycosylated versions of pp-PA83 were produced in *N*. *benthamiana* using either enzymatic deglycosylation *in planta* by co-expression of PNGase F, resulting in pp-dPA83, or site-directed mutagenesis, resulting in pp-PA83M-D or pp-PA83M-Q. These three non-glycosylated versions of pp-PA83, as well as glycosylated pp-PA83, were purified from *N*. *benthamiana* and characterized using immunochemical, biochemical and biophysical methods as described in Materials and Methods. Purified pp-PA83 deglycosylated by treatment with commercial PNGase F *in vitro* was used as a control.

**Fig 1:**
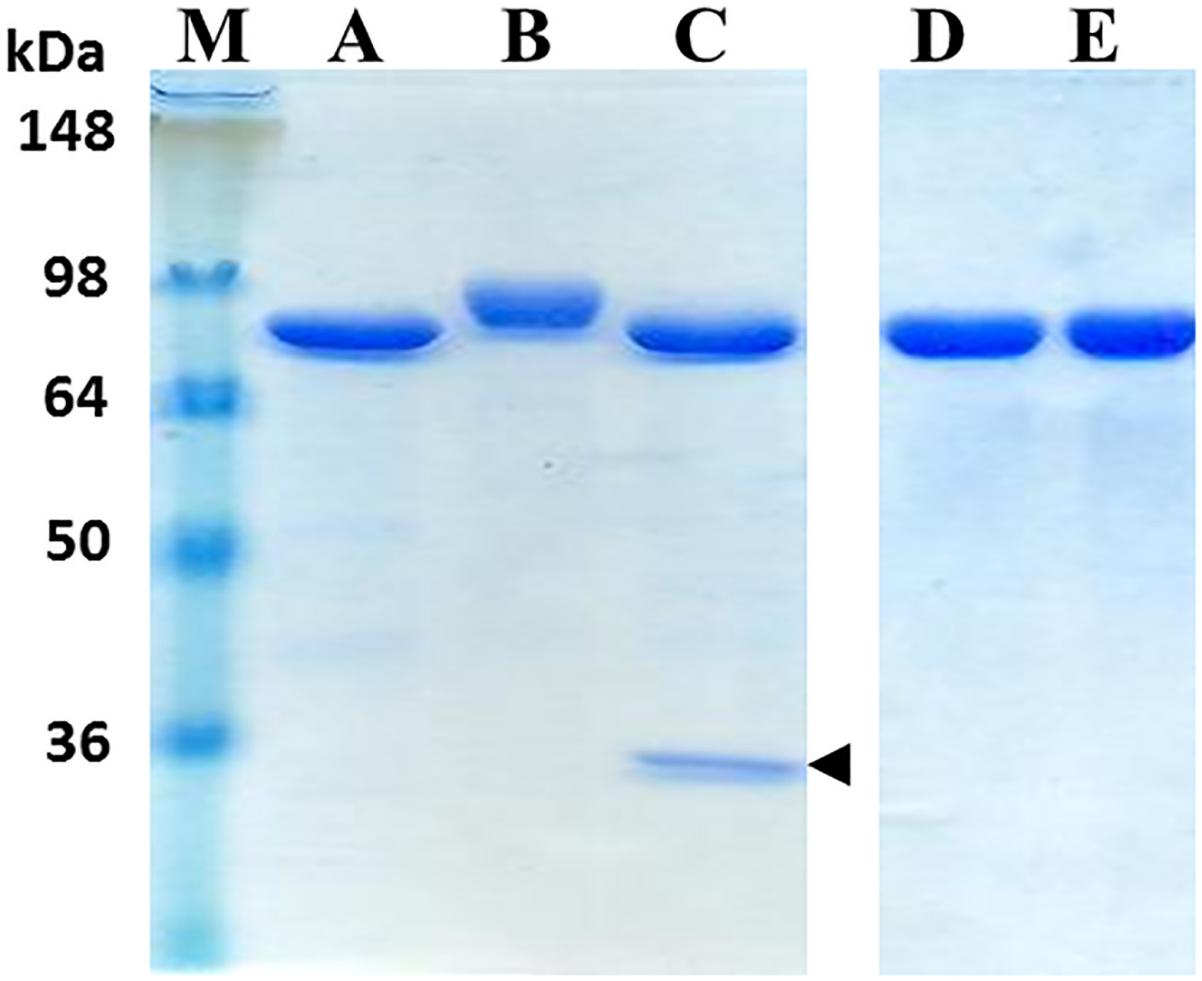
SDS-PAGE analysis of purified pp-PA83 variants. Lanes were loaded with 1.75 μg per lane. M: molecular weight markers, A: pp-dPA83 (*in vivo* deglycosylated), B: glycosylated pp-PA83, C: *in vitro* deglycosylated pp-PA83, D: pp-PA83M-Q mutant, E: pp-PA83M-D mutant. The arrowhead in lane C indicates migration of PNGase F.

**Fig 2:**
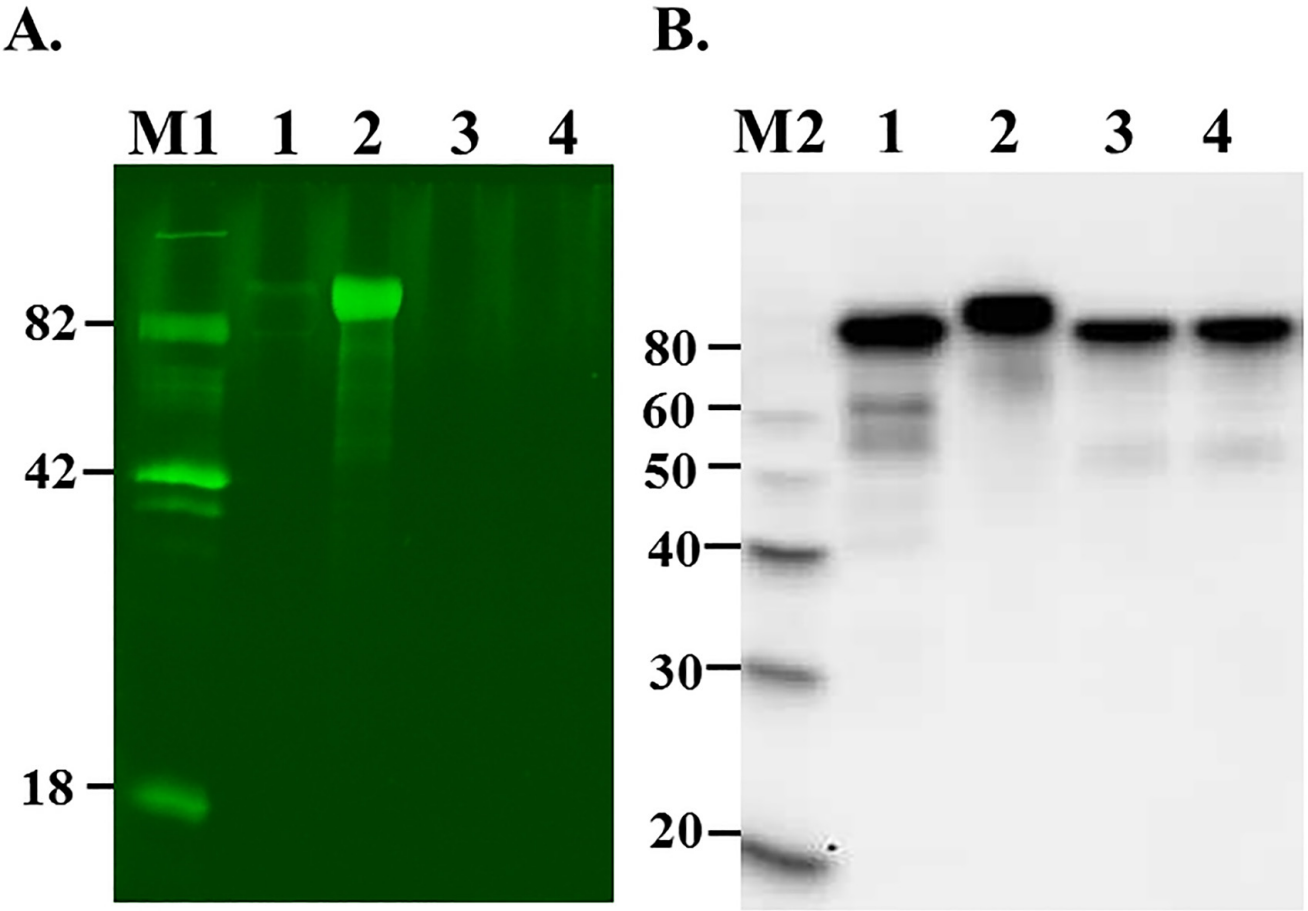
Glycan detection and Western blot analysis of glycosylated and deglycosylated pp-PA83 variants. (A) 0.25 μg of protein from each sample was run on 10% SDS-PAGE followed by in-gel glycan detection using the Pro-Q Emerald 300 glycoprotein staining kit. Stained proteins were visualized by UV illumination. Lanes: 1 –pp-PA83 treated with commercial PNGase F *in vitro*, 2 –glycosylated pp-PA83, 3 –pp-dPA83 treated with commercial PNGase F *in vitro*, 4 –pp-dPA83. (B) Western blot analysis of the same samples using an anti-4xHis tag mAb. M1: CandyCane glycoprotein molecular weight standards (Molecular Probes), 250 ng of each protein per lane; M2: MagicMark XP Western Protein Standard (Invitrogen, Grand Island, NY).

The results of SDS-PAGE analysis of purified pp-PA83 variants showed approximately 90% purity and a shift in the molecular weight of the pp-PA83 protein following deglycosylation and for the mutant variants of pp-PA83 (Fig 1). The absence of glycans on the *in vivo* deglycosylated pp-PA83 protein (pp-dPA83) was confirmed using the glycan-specific stain Pro-Q Emerald 300. Notably, pp-PA83 stains strongly for the presence of glycans (Fig 2A, lane 2) prior to treatment with PNGase F, but shows little staining after PNGase F treatment (Fig 2A, lane 1). No glycan-specific staining was observed in the pp-dPA83 with or without *in vitro* PNGase F treatment (Fig 2A, lanes 3 and 4). The presence of protein was confirmed by Western blot analysis (Fig 2B).

### Stability Assessments of Different Variants of PA83

**Fig 3:**
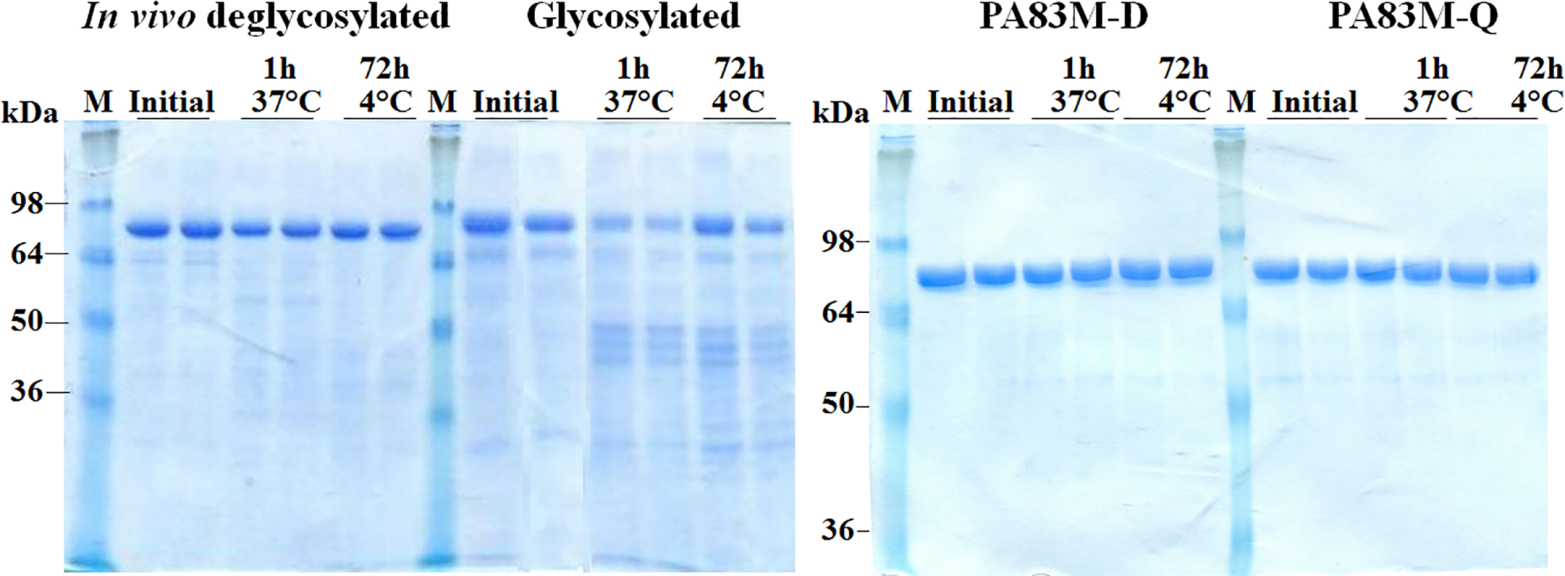
Stability of glycosylated and deglycosylated pp-PA83 variants. pp-PA83 variants were stored for 1 hour at 37°C or for 72 hours at 4°C and analyzed by SDS-PAGE. M: molecular weight markers. Lanes were loaded with ~0.7 μg per lane for *in vivo* deglycosylated pp-PA83 (pp-dPA83) and glycosylated pp-PA83 and with ~1.6 μg per lane for the pp-PA83M-D and pp-PA83M-Q mutated versions.

The stability of glycosylated pp-PA83, *in vivo* deglycosylated pp-PA83 protein (pp-dPA83) and the mutated versions of PA83 (PA83M-Q and PA83M-D) were examined after incubation at 37°C for 1 hour and at 4°C for 72 hours. Analysis by SDS-PAGE showed the predominant form of glycosylated pp-PA83 degrading by more than 70% after incubation at 37°C for 1 hour, whereas pp-dPA83 showed only approximately 30% degradation (Fig 3). By comparison, PA83M-Q and PA83M-D appeared stable (Fig 3). pp-dPA83 also showed better stability than glycosylated pp-PA83 after 72-hours of storage at 4°C (Fig 3), with pp-dPA83 showing a 15% reduction in main band intensity, compared to glycosylated pp-PA83 showing a 50% reduction. These results demonstrate that the deglycosylated forms of pp-PA83 are more stable than glycosylated pp-PA83.

### Affinity of pp-mAb^PANG^ to Different pp-PA83 Variants

Association and dissociation constants between a non-glycosylated plant-produced human mAb against PA (pp-mAb^PANG^) [22] and the various pp-PA83 antigens were assessed by BLItz analysis (Table 1). pp-mAb^PANG^ showed similar binding (dissociation constant) to all pp-PA83 versions, with approximately 3-fold stronger binding for the pp-PA83M-D mutant. The asparagine to aspartic acid mutant showed the most stable complex (lowest off-rate [k_off_]), while having an on-rate (k_on_) in the range of the glycosylated pp-PA83, and thus had the best affinity (K_D_).

**Table 1:** Binding activity analysis of pp-mAb^PANG^ to pp-PA83 variants.

Protein	Treatment	Glycosylated	k_on_ (1/Ms)	k_off_ (1/s)	K_D_^a^(1/M)
pp-PA83	none	Yes	1.37 x 10^5^	1.87 x 10^−3^	1.36 x 10^−8^
pp-PA83	*in vitro* PNGase F	No	1.45 x 10^5^	2.22 x 10^−3^	1.53 x 10^−8^
pp-dPA83	*in vivo* PNGase F	No	6.86 x 10^4^	9.94 x 10^−4^	1.45 x 10^−8^
pp-PA83M-D	mutant	No	1.34 x 10^5^	5.30 x 10^−4^	3.95 x 10^−9^
pp-PA83M-Q	mutant	No	5.92 x 10^4^	6.16 x 10^−4^	1.04 x 10^−8^

^a^ K_D_ represents a calculated value (k_off_ ÷ k_on_).

### LeTx Formation by pp-PA83 Variants

As demonstrated using a TFA, glycosylated pp-PA83 was not able to combine with LF to form LeTx and induce cell death (Table 2). In contrast, *in vivo* deglycosylated pp-PA83 (pp-dPA83) showed an EC_50_ value similar to the positive control, *Bacillus*-produced rPA. The EC_50_ value induced by *in vitro* deglycosylated pp-PA83 was slightly higher compared to pp-dPA83. In addition, pp-PA83M-Q and pp-PA83M-D, in combination with LF, induced little-to-no cell death, respectively.

**Table 2:** LeTx forming assay results for pp-PA83 variants.

Protein	Treatment	Glycosylated	EC_50_ (ng/mL)
rPA^a^	N/A	No	144
pp-PA83	none	Yes	nd
pp-PA83	*in vitro* PNGase F	No	289
pp-dPA83	*in vivo* PNGase F	No	187
pp-PA83M-D	mutant	No	nd
pp-PA83M-Q	mutant	No	916

^a^ recombinant PA from *B*. *anthracis*

N/A: not applicable

nd: not detected

### Immunogenicity of Glycosylated and Non-glycosylated pp-PA83 Variants in Mice

**Fig 4:**
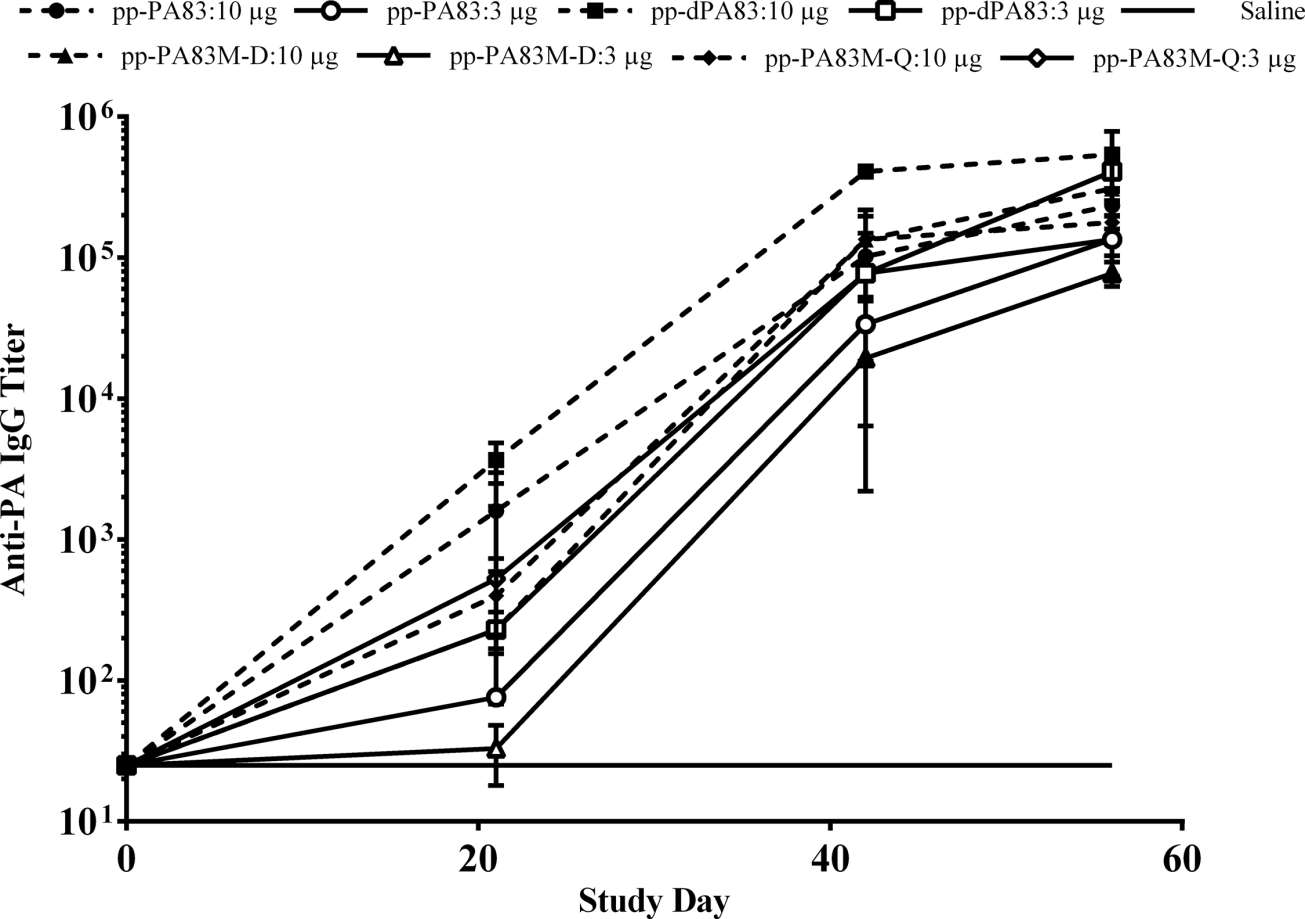
IgG responses in mice elicited by pp-PA83 variants using Alhydrogel as an adjuvant. IgG titers were determined by ELISA in sera collected on multiple days post vaccination from mice immunized with one of the four pp-PA83 variants. Reciprocal serum dilutions that gave a mean OD value greater than 4-fold over background were determined as the end point titers. Data are shown as the geometric mean serum IgG end point titers per group ± standard error of the mean. Statistical significance (*p*<0.05) was calculated using the Mann-Whitney test.

**Fig 5:**
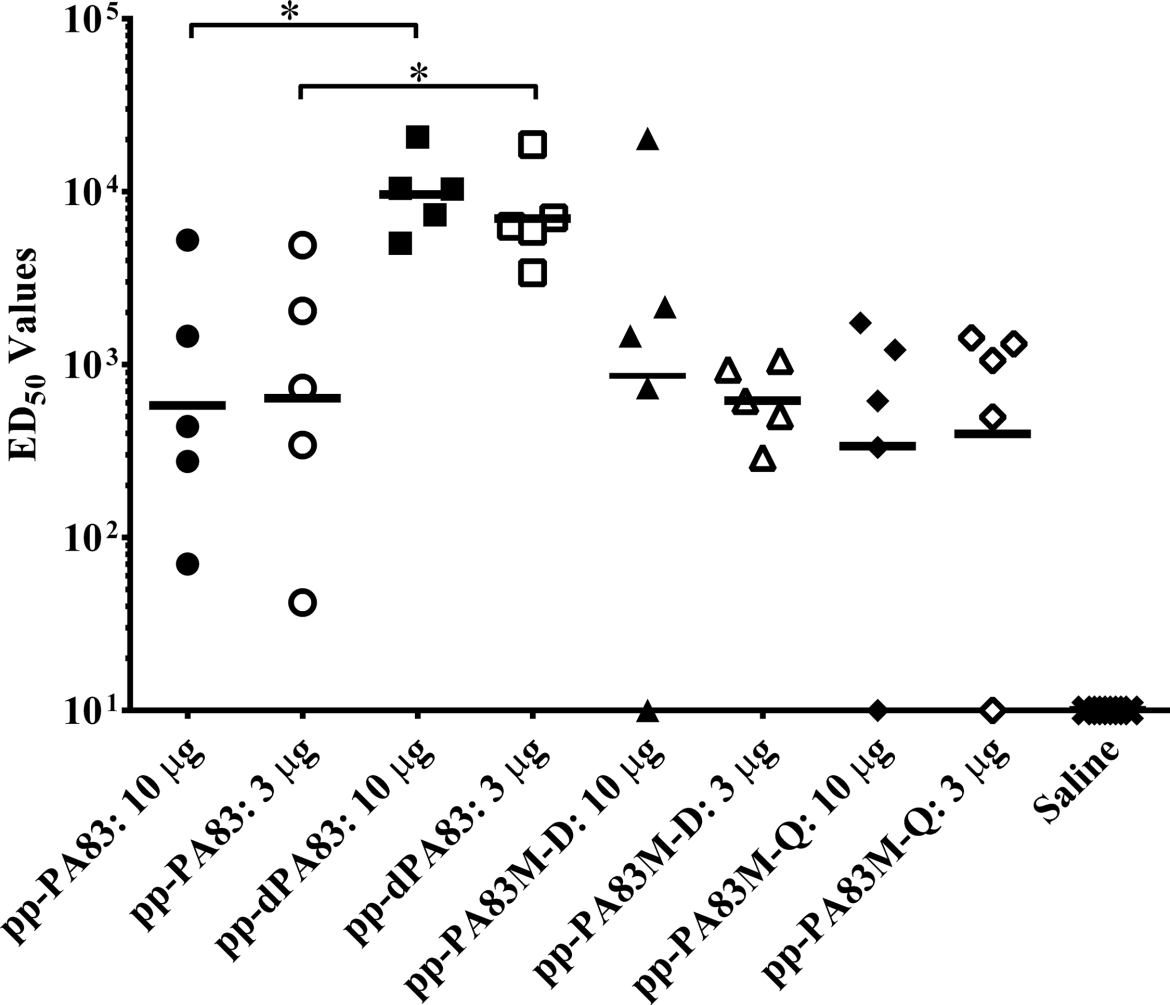
TNA responses elicited by the different variants of pp-PA83 in mice. Mice were immunized on study days 0, 21 and 42 IM with 10 μg (solid symbols) or 3 μg (open symbols) of pp-PA83 protein adsorbed to 0.3% Alhydrogel. Serum samples were collected on study day 56 (post 3^rd^ vaccination) and analyzed for TNA using the LeTx neutralization assay. Data are presented as the ED_50_ of LeTx for the corresponding serum sample with the solid line representing the geometric mean of the ED_50_ values per group of mice. Statistical significance was calculated using the one-way ANOVA and Kruskal-Wallis tests where * = *p*<0.05.

Mice received three doses of different variants of the pp-PA83 protein adsorbed to 0.3% Alhydrogel at three-week intervals (0, 21 and 42 days). Prior to immunization, the activity of pp-PA83 proteins was confirmed in a TFA. Groups of seven-week-old mice (5 animals/group) were immunized IM with either 10 or 3 μg of pp-PA83 variants. Results of the serum analysis on study day 56 showed that mice immunized with the *in vivo* deglycosylated protein (pp-dPA83) developed ~2-3-fold greater PA-specific antibody titers compared to serum from animals immunized with either glycosylated pp-PA83 or the two mutants (Fig 4). This increase in antibody titer generated by the pp-dPA83 vaccine was statistically significant (*p*<0.05) in the 3 μg dose groups. pp-dPA83 also induced significantly higher (~10-fold; *p*<0.05) TNA responses after three doses compared to glycosylated pp-PA83 and either of the two mutants (Fig 5).

**Fig 6:**
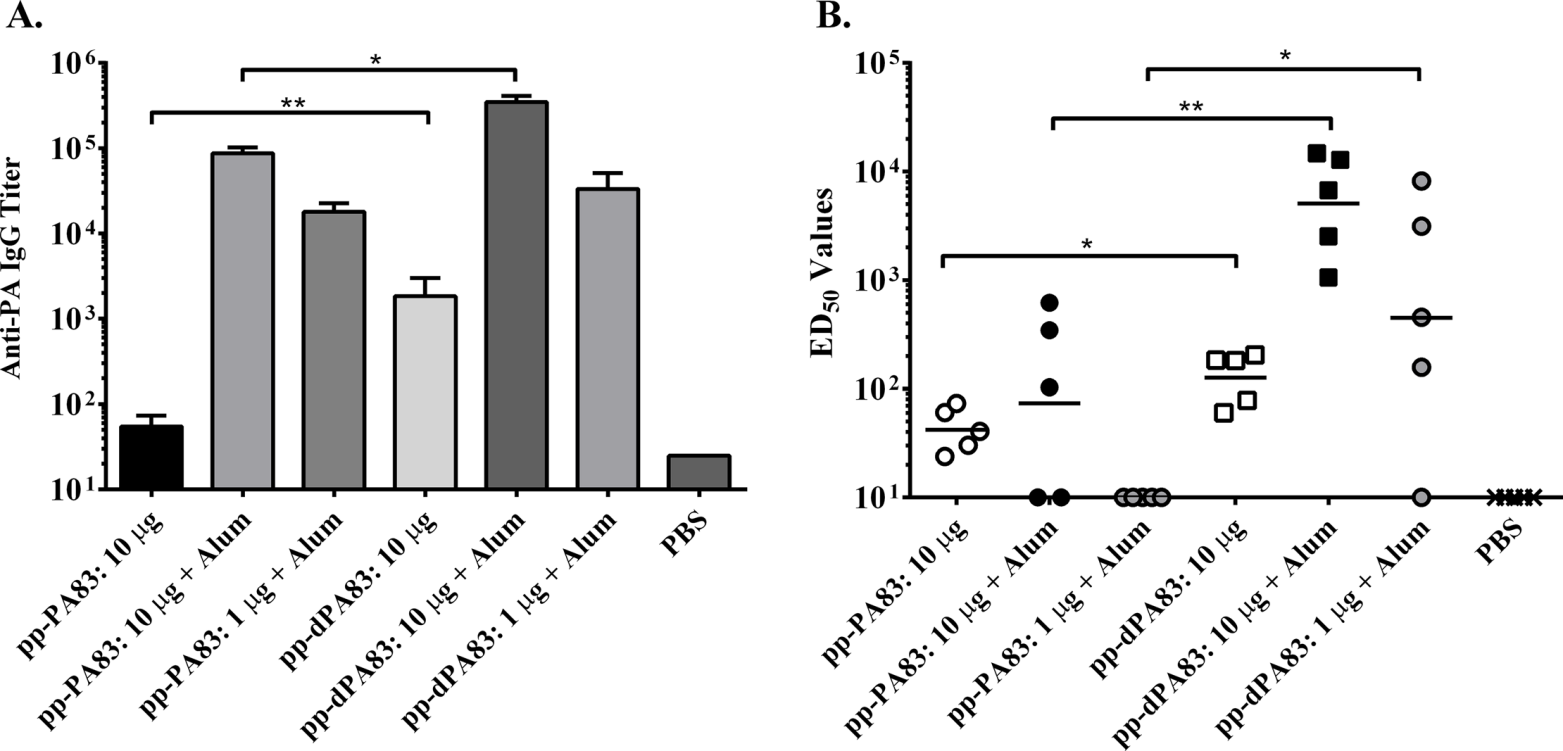
Comparative immunogenicity of pp-PA83 and pp-dPA83 in mice. Mice were immunized on study days 0 and 21 IM with 10 (with or without alum) or 1 μg (with alum) of pp-PA83 or pp-dPA83. Serum samples were collected on study day 42 (post 2^nd^ vaccination) and analyzed for anti-PA IgG responses by ELISA (A) and TNA (B) using the LeTx neutralization assay. ELISA response are presented as the geometric mean serum IgG end point titers per group ± standard error of the mean and TNA data are presented as individual values with the geometric mean of the ED_50_ values per group of mice indicated by the black line in each data set. Statistical significance was calculated using the Mann-Whitney test where * = *p*<0.05 and ** = *p*<0.01.

A second mouse immunogenicity study was conducted comparing pp-PA83 and pp-dPA83 proteins that were produced at the same plant biomass scale and purified using the same methodology to account for any effect host cell impurities may have had on the results of the initial *in vivo* comparison. In this study, groups of eight-week old mice (5 animals/group) were immunized IM with 10 or 1 μg of either pp-PA83 or pp-dPA83. To broaden the parameters of the original comparison, the 10 μg dose was tested with and without Alhydrogel adjuvant, while the 1 μg dose was only tested in the presence of adjuvant. Immunizations were administered on study days 0 and 21 for a total of two doses compared to the three immunizations used in the previous study. Serum was collected on study day 42, three weeks after boost, and analyzed for anti-PA and TNA antibody responses (Fig 6). Assessment of the total PA-specific IgG responses showed that pp-dPA83 was able to induce significantly higher titers of antibody both with (*p*<0.05) and without (*p*<0.01) alum adjuvant at the 10 μg dose, although the magnitude of the responses in all groups was greatly enhanced by the use of adjuvant (Fig 6A). At the 1 μg dose, both antigens elicited equivalent levels of anti-PA IgG. Evaluation of the TNA responses in these animals revealed that at all three conditions (10 μg dose with or without alum adjuvant and 1 μg dose with adjuvant), pp-dPA83 induced significantly (*p*<0.05) higher levels of functional antibody titers (Fig 6B).

## Discussion

*B*. *anthracis*, the causative agent of anthrax, has been targeted for use as a biological weapon. The availability of safe, efficient and easily delivered vaccines against a pathogen like anthrax is of paramount importance to protect civilian populations and military personnel. Prophylactic vaccination is the first line of defense against anthrax with therapeutic vaccination and countermeasure agents such as mAbs and antibiotics as potential post exposure treatment options. As shown in animal models, protective immunity against anthrax correlates with production of antibodies against PA that neutralize the activity of the two anthrax exotoxins [25–27]. Therefore, PA has become the main target for anthrax vaccine development. The only approved anthrax vaccine in the U.S. is BioThrax^®^ (Anthrax Vaccine Adsorbed) indicated for individuals at high risk of exposure. However, due to the limitations of BioThrax^®^, such as undefined composition, lot-to-lot variability, multiple-dose administrations and safety concerns, efforts are being made to develop a safer and effective anthrax vaccine that can be administered in fewer doses.

Recombinant subunit vaccines provide a promising alternative to pathogen-derived material. They are inherently safe and well defined and their production can be highly standardized to ensure batch-to-batch consistency. Several experimental rPA-based anthrax vaccines have been produced using *Escherichia coli* cell expression systems (SparVax^TM^, PreviThrax^TM^ and NuThrax^TM^). These vaccine candidates have been shown to elicit serum anti-PA and TNA antibodies and to confer protection against lethal aerosolized anthrax challenge in several animal models, including rabbits and non-human primates [5,25,28]. In addition, several of these vaccine candidates have been shown to be safe and immunogenic in humans, despite differences in the amount of Alhydrogel adjuvant used and the number of doses administered [7,29].

Plants have emerged as a suitable alternative platform for the production of recombinant vaccine antigens, mAbs and therapeutic proteins. Plant-based expression systems are cost-efficient and highly scalable, have high production capacity and do not harbor mammalian pathogens. The strategies for target gene delivery and protein expression in plants include engineering of transgenic or transplastomic plants and plant systems for transient expression. Several efforts have been made to produce immunogenic rPA in plant systems through stable integration of the PA gene into host chloroplast genome [30–32]. For example, rPA produced in tobacco chloroplasts [30] elicited high anti-PA IgG titers (up to 1:320,000) in mouse serum and conferred 100% survival on mice upon challenge with lethal doses of LeTx, the same protection level as provided by *B*. *anthracis*-derived PA [30]. Therefore, tobacco chloroplasts might be a suitable system for producing rPA. However, while expression levels of vaccine antigen are enhanced in this system, the transplastomic approach has relatively long lead times due to the time required for developing transformed lines. As an alternative for more rapid responses, the focus has turned to transient expression of antigens in plants based on the replication of recombinant plant viral vectors. This expression approach is now widely used and allows for the production of large quantities of target proteins within a short time frame [33–37], a particularly attractive feature in the case of pathogens that may be used for bioweapons and for epidemics. Some recombinant proteins, including vaccine antigens, produced in transient plant systems have reached clinical or advanced pre-clinical stages of development (reviewed in [38,39]).

However, the ability of eukaryotic expression systems, including plants, to glycosylate proteins may be undesirable for some proteins that do not carry N-linked glycans in the native hosts, including PA of *B*. *anthracis*. In previous studies we produced glycosylated full-length PA (PA83) using a hybrid vector-based transient plant expression system. Although this plant-produced glycosylated PA83 (pp-PA83) elicited high TNA titers in mice and rabbits after two vaccine administrations with Alhydrogel and protected rabbits from a lethal aerosolized challenge infection [15], further improvements in the area of enhanced immunogenicity and stability were desired. Candidate vaccines that could increase vaccine stability while enhancing or accelerating the host’s ability to generate protective immune responses could decrease costs while maximizing efficacy, two desirable features to stockpiling a vaccine and battling an emerging pathogen.

To address this need, FhCMB redesigned how the PA83 target protein was produced *in planta*. During characterization of the pp-PA83 vaccine, it was noted that this molecule could not form LeTx *in vitro*. We hypothesized that this may be due to aberrant glycosylation, possibly resulting in either incorrect protein folding or the masking of critical neutralizing epitopes. Therefore, a recently developed strategy for enzymatically deglycosylating proteins *in planta* was applied for the pp-PA83 target. In this strategy, bacterial PNGase F is co-expressed with the target of interest resulting in non-N-glycosylated recombinant protein production that better represents the wild-type antigen [16]. Another successful application of this technology was shown with a malaria vaccine candidate based on the Pfs48/45 antigen and produced in *N*. *benthamiana* in a non-N-glycosylated form. This non-glycosylated antigen was recognized 2- to 6-fold better than the glycosylated forms of Pfs48/45 when assessed by ELISA using critical epitope-specific mAbs I, III (conformation-specific) and V [16].

In the current study, we successfully applied this PNGase F deglycosylation approach to generate non-glycosylated rPA (pp-dPA83) of *B*. *anthracis*. When purified pp-dPA83 was assessed for stability, it appeared to be more stable than the glycosylated counterpart (pp-PA83). In addition, we demonstrated that pp-dPA83 elicited significantly higher levels of TNA titers in immunized mice compared with glycosylated pp-PA83. To assess whether these differences in stability and immunogenicity could be explained by glycosylation status, we also produced non-glycosylated forms of pp-PA83 using *in vitro* deglycosylation with commercial PNGase F or by mutating potential N-glycosylated sites of pp-PA83. The LeTx neutralization activity and immunogenicity of *in vitro* deglycosylated pp-PA83 and the pp-PA83M-D mutant were shown to be much lower than those of pp-dPA83. This suggests that *in vitro* activity of pp-dPA83 may reflect correct protein folding with the non-active versions potentially misfolded, regardless of their glycosylation state. This was further investigated through antibody binding assessment by BLItz. The results showed similar affinities of all rPA versions to the toxin neutralizing mAb pp-mAb^PANG^. Interestingly, the pp-PA83M-D mutant had the best affinity constant (K_D_). Both pp-dPA83 and pp-PA83M-D should have identical amino acid sequences, since during glycan removal by PNGase F the modified asparagine residue is converted to aspartic acid, but differences in their affinity to pp-mAb^PANG^ and their ability to form LeTx and generate toxin neutralizing serum responses also suggest potential differences in protein folding. The difference in ranking of pp-dPA83 and pp-PA83M-D in regard to affinity versus TNA indicates that a mAb such as pp-mAb^PANG^ may not be the best tool for comparison of potency, particularly since it only represents a single functional epitope. Thus, *in vivo* enzymatic deglycosylation can be a robust, cost-effective strategy to produce rPA in a non-glycosylated form, which may be important for preserving native conformation and biological activity. The enhanced biological activity of pp-dPA83 could provide advantages as a vaccine candidate over the glycosylated version, such as dose sparing and accelerated immunogenicity, which in turn could decrease the cost of the vaccine while enhancing vaccine recipient compliance. While additional formulation, pre-clinical and stability testing of this vaccine candidate are necessary, the results of this study suggest the potential of pp-dPA83 as an anthrax vaccine candidate and support its further characterization and early stage clinical development.
